# Impact of prenatal cold stress on placental physiology, inflammatory response, and apoptosis in rats

**DOI:** 10.18632/oncotarget.23257

**Published:** 2017-12-14

**Authors:** Shuai Lian, Jingru Guo, Lipeng Wang, Wenjie Li, Jianfa Wang, Hong Ji, Fanzhi Kong, Bin Xu, Shize Li, Huanmin Yang

**Affiliations:** ^1^ College of Animal Science and Veterinary Medicine, Heilongjiang Bayi Agricultural University, Daqing 163319, P. R. China

**Keywords:** prenatal cold stress, placental, inflammatory, apoptosis, NF-κB

## Abstract

Prenatal cold stress is one of the earliest factors affecting mammalian health, and is associated with neonatal growth retardation and immune dysfunction, thus increasing disease susceptibility. The mechanisms underlying these observations remain unclear; hence, the objective of this study was to elucidate placental responses to cold stress. 60 maternal rats were randomly allocated to either stressed (n = 30) or non-stressed (control, n = 30) treatment conditions and 30 pubs (n=15) were used for the pups analysis. We found that maternal exposure to cold stress resulted in decreased body temperature, increased food intake without body weight gain, and a high level of plasma corticosterone (CORT) between gestational day (GD) 14 and GD21. In addition, gestation cold stress induced the placental expression of heat shock protein 70 (HSP70), IκBα, glucocorticoid receptor (GR), mineralocorticoid receptor (MR), 11β-hydroxysteroid dehydrogenase 2 (11β-HSD2), interferon (IFN) regulatory factor 3 (IRF3), Caspase-3 proteins and altered the ratio of B-cell lymphoma-extra large (Bcl-xL) to Bcl-associated x (Bax) proteins on gestational GD15, GD17, GD19, and GD21, also resulted in the production of interleukin (IL)-1β. Next, gestational cold stress provoked a decrease in plasma GH levels of 21-day-old offspring, and the body weights of offspring were have no differences from postnatal day (PD) 1–21. Taken together, our results indicate that gestational cold stress induces placental apoptosis and the activation of NF-kB via HSP70/TLR4/NF-κB signaling pathways in the placenta, these changes may affect placental function and fetus development.

## INTRODUCTION

Stress is part of normal life, experienced in biological systems, which triggers many health conditions and disorders [1]. Some stressors such as physical exercise and creative activity are usually considered healthy [2]. However, when exposure to stress is chronic, prolonged activation of the stress response may become maladaptive and have adverse consequences for the individual [3]. Cold stress include prenatal cold stress is one of these stressors, which is a major factor that can negatively affect the growth and production of all livestock species in north frigid area [4]. During this condition, energy harvesting during acute cold contributes to maintaining the body temperature. However, little is known about the effect of temperature and the feed intake changes on placental metabolism/function and that of the embryo/fetus during pregnancy cold stress, the underlying mechanisms behind these impacts remain unclear.

Stress could cause a persistent hyperactivity of the HPA axis and thus elevated glucocorticoid levels, glucocorticoids could pass through the placental barrier and reaches the fetus [5]. The placenta plays a pivotal role in the acceptance of the fetal-placental unit by the maternal immune system, and forms a barrier to maternal glucocorticoids [6]. A placental enzyme, 11β-HSD2, normally protects the fetus from relatively high levels of maternal glucocorticoids, by inactivating approximately 80–90 % of maternal cortisol [5]. However, this protection is not complete: one study has clearly demonstrated that maternal stress results in increased circulating cortisol in guinea pig fetuses [7], endogenous glucocorticoids bind to both glucocorticoid and mineralocorticoid receptors (GR and MR, respectively), which themselves are subject to autoregulation, and this MR/GR dual-receptor system regulates negative feedback of the hypothalamic–pituitary–adrenal (HPA) axis, which is important for homeostatic control [8]. In the fetal circulation and in particular the fetal brain, maternal cortisol can perturb the development of the fetal HPA axis during vulnerable periods by resetting the set point of the HPA axis’s negative feedback mechanism, resulting in a long-lasting or even permanent change in HPA axis activity in postnatal life [9].

Heat shock protein 70 (HSP70), plays an important role in protein trafficking and the refolding of denatured proteins during stress, which can activate the TLR4 signaling pathway and subsequently induce proinflammatory cytokine production via NF-κB and IRF-3 signal pathways [10, 11]. But it is not clear the hypothesis behind to the effects of cold stress on HSP70 and proinflammatory cytokine production. Moreover, apoptosis can be triggered by a variety of factors, such as reactive oxidative stress [12] and inflammatory response [13]. However, research on the effects of cold stress in gestation on placental apoptosis is limited. The present study focused on the maternal response to prenatal cold stress and the data from offspring will be included in future studies.

## RESULTS

### Body temperature, feed intake, body weight gain and gestational length

**Figure 1:**
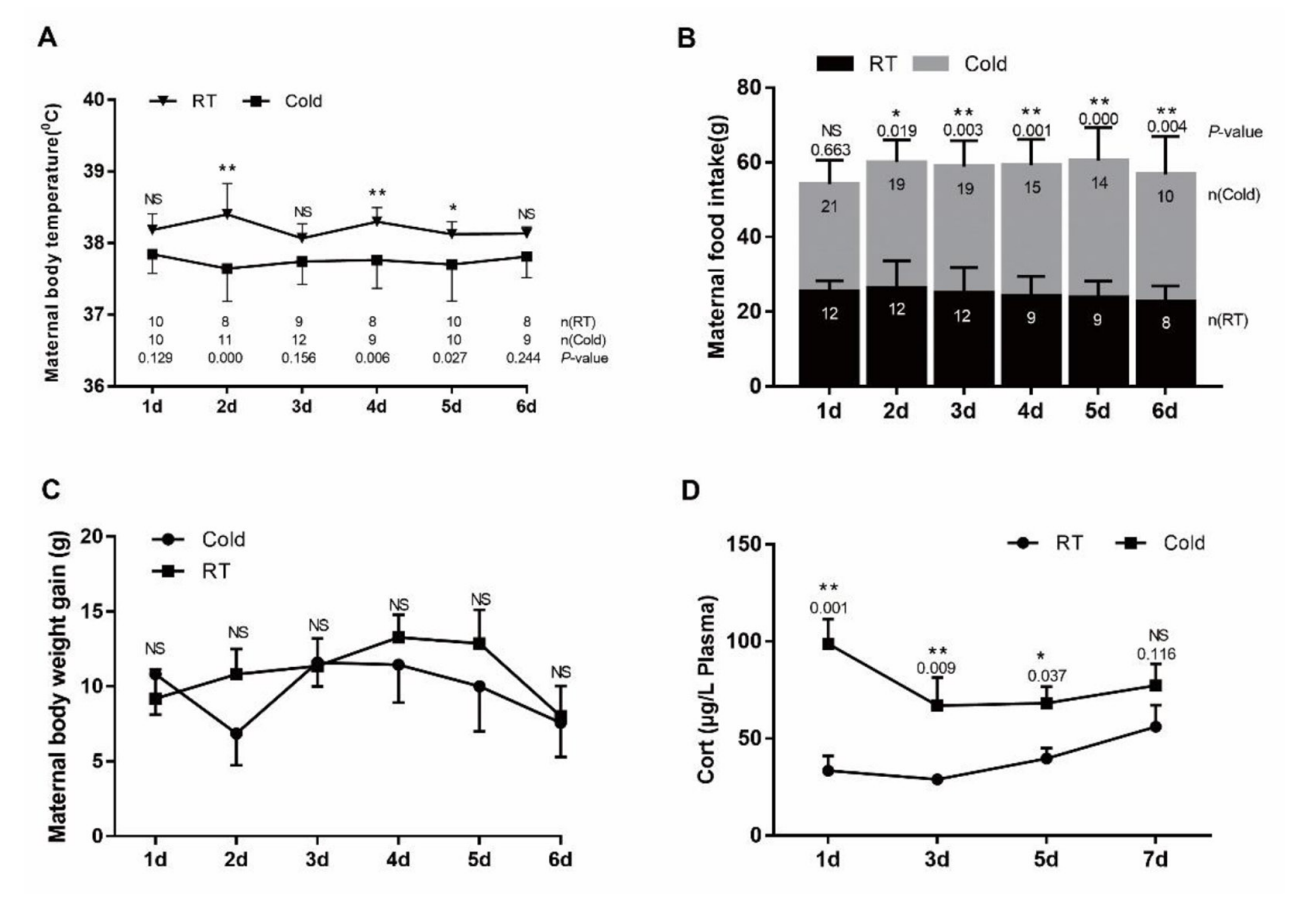
Impact of prenatal cold stress on maternal body temperature, food intake, and plasma CORT levels **(A)** Rectal body temperature before or after cold stress. **(B)** Maternal food intake before or after cold stress during gestation. **(C)** Maternal body weight gain before or after cold stress during gestation. **(D)** Maternal plasma CORT levels (n = 6). The data are shown as mean ± SD, statistically significant differences are indicated: ^*^P<0.05, ^**^P < 0.01.

Acute cold exposure led to significant body temperature decreases in the stressed group compared with the control group (Figure 1A). Feed intake level for 2d-6d was significantly different between the stressed and control groups (Figure 1B, *P* < 0.019). Those rats subjected to 2–6 days of cold stress consumed significantly more feed than those cold-stressed for one day. Body weight gain data for the pregnant rats is represented in Figure 1C. Although body weight gains in the stressed groups were numerically lower than those of the control groups, the differences did not reach statistical significance (*P*>0.05).

### Maternal plasma corticosterone levels

Stress causing activation of the HPA axis results in increased corticosterone (CORT) concentrations, driven by increased secretion of ACTH. Prenatal ACTH has been assumed to increase maternal CORT levels [14]. During gestational cold stress, we found increased corticosterone plasma levels after 1 (*P*=0.001), 3 (*P*=0.009) and 5 (*P*=0.037) days of cold stress. At day 6 of cold stress, plasma corticosterone levels were slightly decreased, but still higher than those in the control groups, and they remained elevated until the end of gestation (Figure 1D).

### Expression of CD4 and CD8 antigen on T cells in peripheral blood

**Figure 2:**
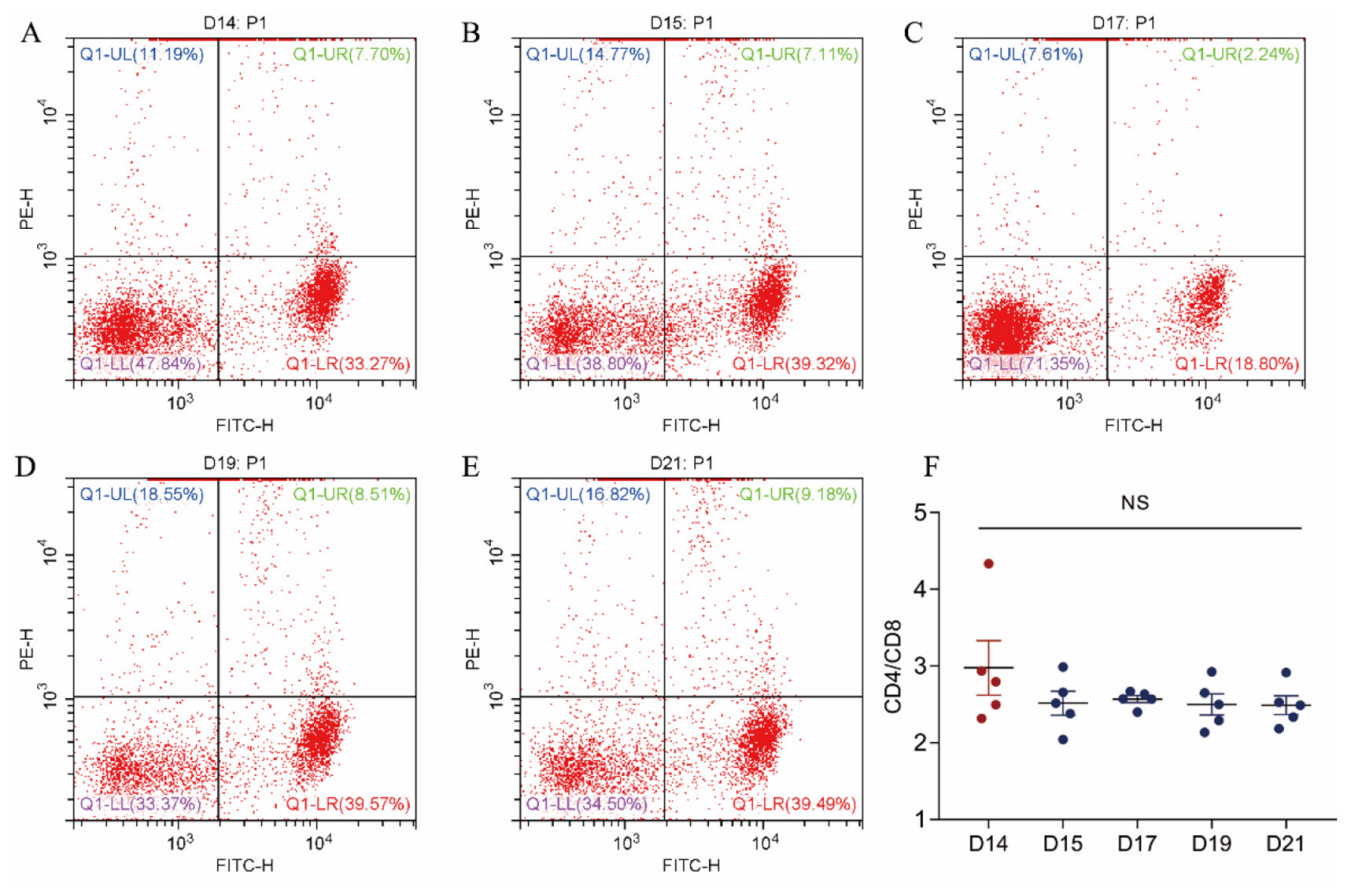
The effect of cold stress on the expression of CD4 and CD8 antigen on T cells in peripheral blood **(A, B, C, D, E)** Representative figures from GD14 to GD21 flow cytometry analyses. **(F)** Analyzed data of CD4/CD8 ratio. Values are expressed as means ± SD (n = 5).

CD4/CD8 analysis (Figure 2) revealed that the CD4/CD8 ratio in the control group was numerically higher than those of the stressed groups, but no the difference was not significant (control 2.97 ± 0.3653; stressed 2.547 ± 0.1886, 2.48 ± 0.055, 2.512 ± 0.1853, 2.485 ± 0.1592).

### Prenatal cold stress activated the placental HSP70/TLR4/NF-κB signaling pathway

**Figure 3:**
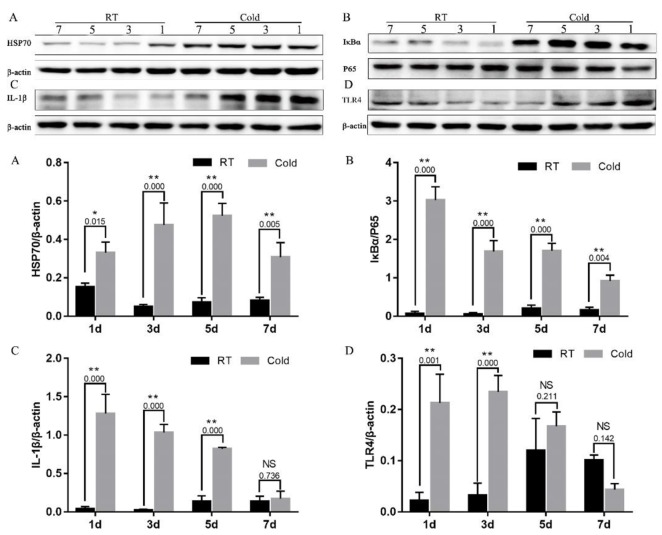
Impact of prenatal cold stress on the activation of the HSP70/TLR4/NF-κB signaling pathway in placenta **(A, D)** HSP70 and TLR4 protein levels in placenta. **(B, C)** Levels of IκB, P65, and IL-1β. The data are presented as mean ± SD (n = 5). These are cropped blots, full-length blots are presented in Supplementary Figure 1. Statistically significant differences are indicated: ^*^P < 0.05, ^**^P < 0.01.

To further investigate the prenatal stress inflammatory mechanism, we examined the HSP70/TLR4/NF-κB signaling pathway, by measuring placental HSP70, TLR4, IκB, IL-1β, and P65 protein levels. As shown in Figure 3, prenatal cold stress enhanced HSP70 (Figure 3A, *P* < 0.015) and IκBα expression (Figure 3B, *P* < 0.004). We also demonstrated that IL-1β levels increased after cold-stress periods of 1, 3, and 5 days (Figure 3C, *P*=0.000), and that they decreased after 7 days of cold stress (Figure 3C, *P*=0.726). Interestingly, placental TLR4 expression followed a similar trend after cold-stress periods of 1 (*P*=0.001) and 3 (*P*=0.000) days (Figure 3D), these results were possible followed the CORT levels. Our results indicate that prenatal cold stress induced activation of NF-kB via HSP70/TLR4/NF-κB signaling pathways in the placenta.

### Prenatal cold stress induced expression of GR, MR, 11β-HSD2, and IRF3 in placenta

**Figure 4:**
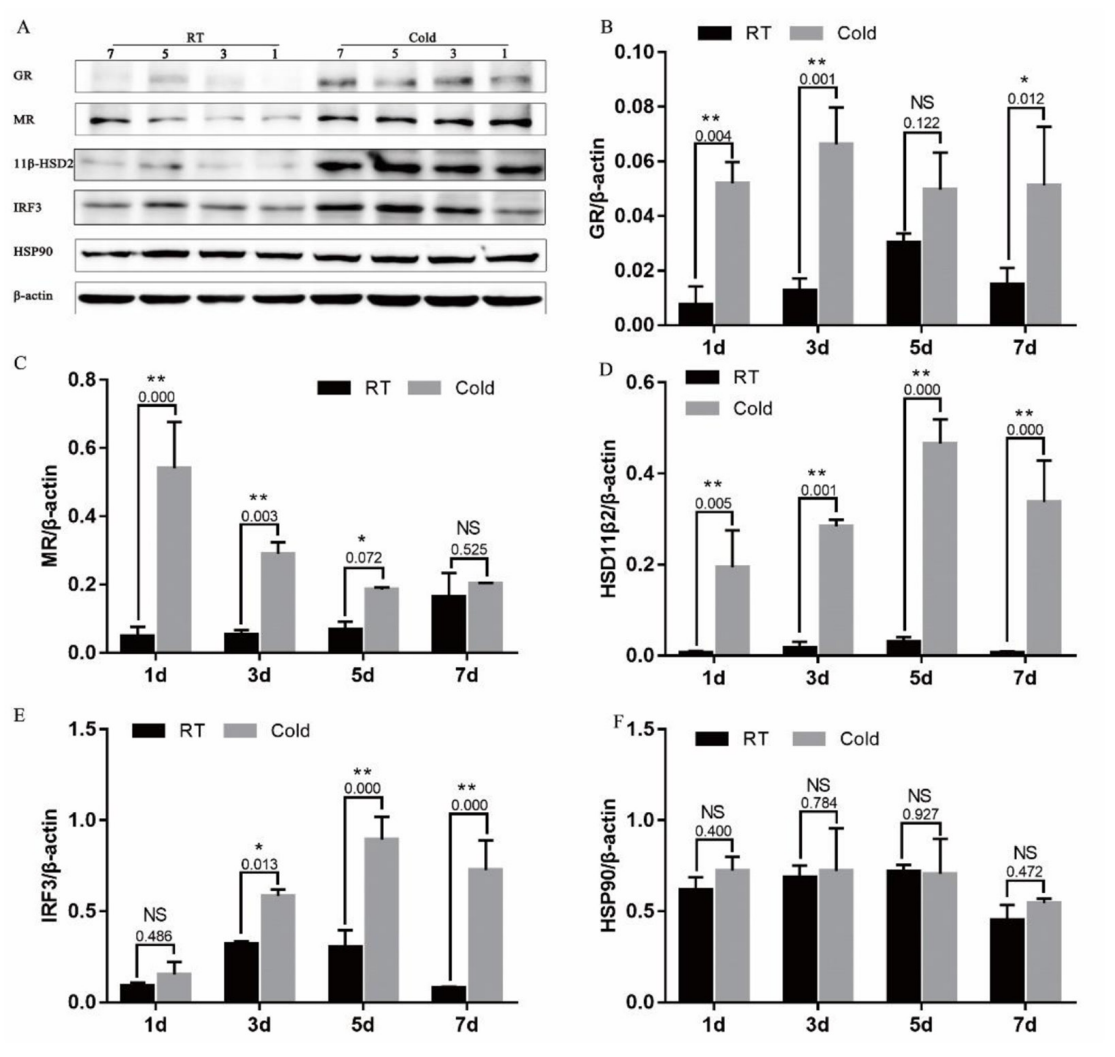
Effect of prenatal cold stress on the expression of GR, MR, 11β-HSD2, and IRF3 proteins These are cropped blots, full-length blots are presented in Supplementary Figure 2. The data are presented as mean ± SD (n = 5). Statistically significant differences are indicated: ^*^P < 0.05, ^**^P < 0.01.

As shown in Figure 4, prenatal cold stress had a significant effect on grey intensity values of GR (Figure 4B) after 1 (*P*=0.004), 3 (*P*<0.001) and 7 (*P*<0.012) days of cold stress, MR (Figure 4C) after 1 (*P*=0.000), 3 (*P*=0.003) and 5 (*P*=0.072) days of cold stress, and 11β-HSD2 (Figure 4D, *P* < 0.005). Furthermore, we measured levels of IRF3, an interferon regulatory factor, and levels of HSP90. There was no effect on IRF3 of cold stress 1d (*P*=0.486), as the duration of cold stress was extended, placental IRF3 expression significantly increased (Figure 4E, *P* < 0.013). However, cold stress had no significant effect on HSP90 expression (Figure 4F, *P*>0.400).

### Bcl-xL/Bax ratio and Caspase-3 protein expression in placenta

**Figure 5:**
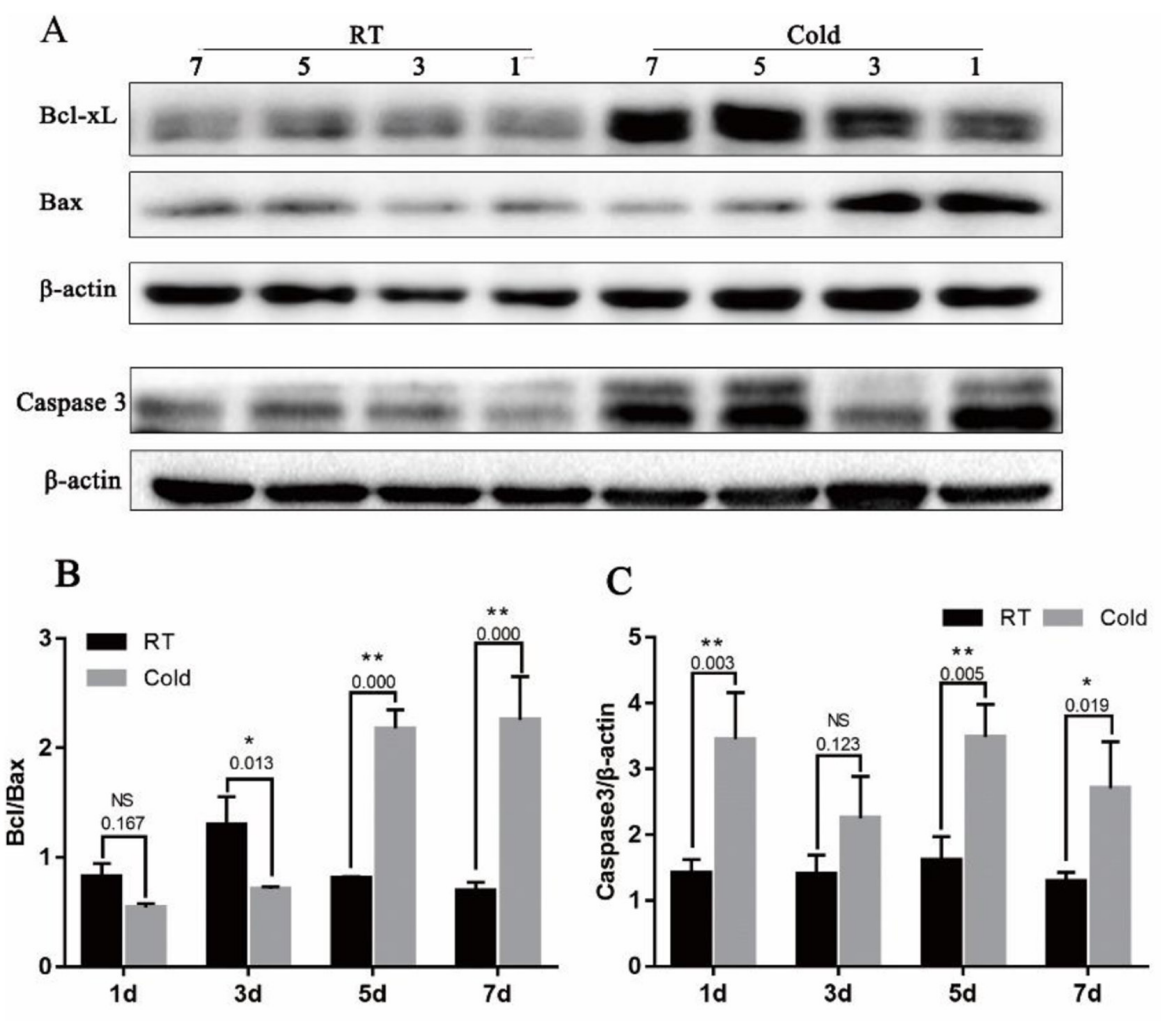
Effect of prenatal cold stress on the expression of Bcl-xL, Bax and Caspase3 proteins **(A)** These are cropped blots, full-length blots are presented in Supplementary Figure 3. **(B)** Bcl-xL/Bax ratio was determined by western blot. **(C)** Caspase3 protein levels in placenta. The data are presented as mean ± SD (n = 5). Statistically significant differences are indicated: ^*^P < 0.01, ^**^P < 0.01.

In addition, to determine the influence of cold stress on cell apoptosis, we examined the expression of Bcl-xL (an anti-apoptotic protein), Bax (a pro-apoptotic protein) and Caspase-3 (a key enzyme in the mitochondria-dependent apoptosis pathway). As shown in Figure 5, The Bcl-xL/Bax expression ratio reached a maximum value after cold stress of 5 d and 7 d (Figure 4B, *P* =0.000). In addition, Caspase-3 protein expression was increased after cold-stress, especially in the periods of 1 (*P*=0.003), 5 (*P*=0.005) and 7 days (*P* = 0.019).

### The pups’ plasma GH and body weight

**Figure 6:**
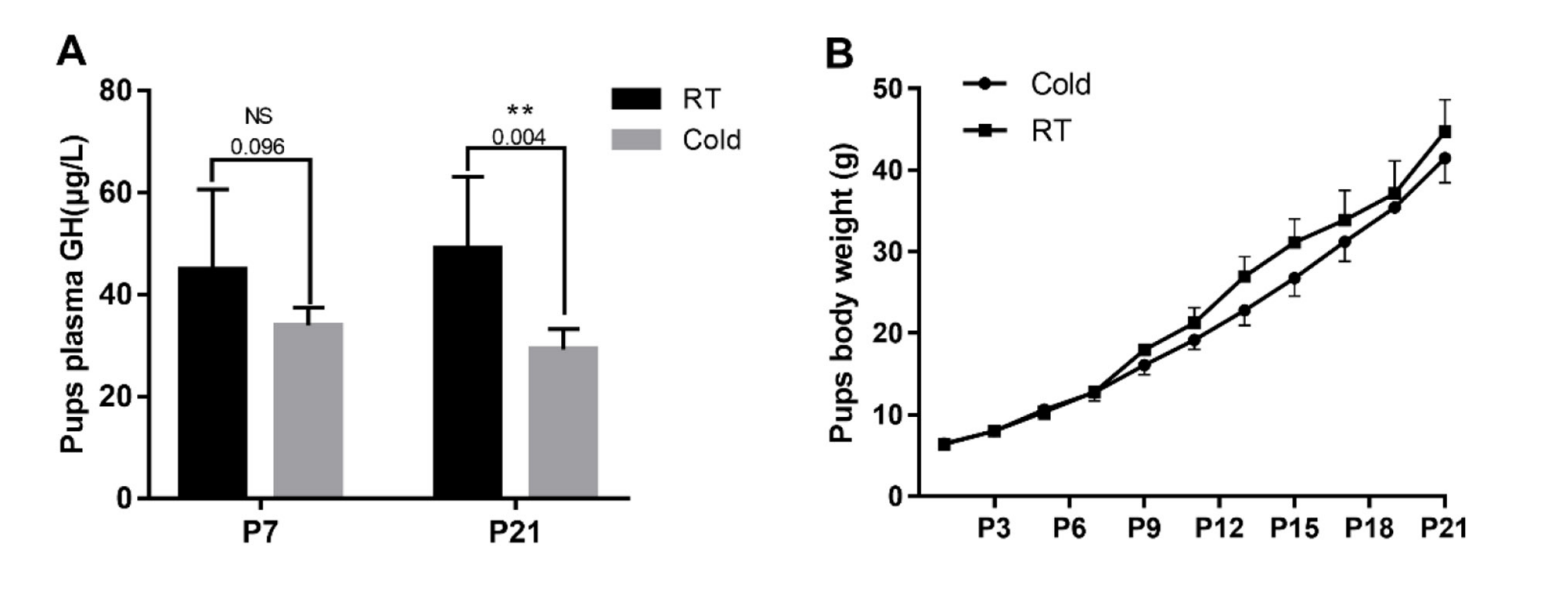
Prenatal cold stress decreased plasma GH levels and the body weight of offspring **(A)** ELISA detection for 7 and 21 d old pups’ plasma GH levels (n=6-10). **(B)** Pups’ body weight growth curve (from postnatal days 1 to 21) (n=30). The data are presented as mean ± SD. Statistically significant differences are indicated: ^*^P < 0.05, ^**^P < 0.01.

To evaluate the effect of gestational cold stress on the offspring, we weighed the pups from 1 to 21 d after birth and tested their plasma growth hormone levels on days 7 and 21. We demonstrated that the body weight began to appear difference from 7^th^ days, however, there were no significant differences between the cold stress and control groups (Figure 6B). Interestingly, gestational cold stress provoked a decrease in plasma GH levels of 21-day-old offspring (Figure 6A, *P* = 0.004).

## DISCUSSION

Stress has been demonstrated to impact virtually every organ in the body, even in the fetus. In this study, we provide evidence that prenatal cold stress exerts a significant effect on the placenta, which is associated with the HSP70/TLR4/NF-κB signaling pathway, a corresponding reduction in body temperature, and other factors.

Rectal temperature is considered a good indicator of core body temperature because of its close proximity to the abdominal cavity and longer equilibrium period [15]. The present study shows that prenatal cold stress induced a decrease in prenatal rats’ body temperature and induced an increase in the food intake of gravid rats, without body weight gain (Figure 1C) and the fetus weights were not proportional to the feed intake (Figure 6B). These data suggest that most of the energy harvesting during acute cold mainly contributes to maintaining the body temperature, fewer flow to the placenta/fetus.

Prenatal cold stress induced a low CD4/CD8 ratio in maternal blood. CD4 and CD8 are members of the immunoglobulin superfamily. CD4 potentiates the inflammatory or humoral immune response through the actions of Th1 and Th2 cells, respectively [16], interacts with the MHC class II molecule, and is involved in T cell activation. CD8 T cells combat viral infections [17], having an antigen co-receptor that interacts with MHC class I molecules on antigen-presenting cells or epithelial cells. A low CD4/CD8 ratio has been identified in the general population as a hallmark of immunosenescence and a surrogate of all-cause mortality; thus, the CD4/CD8 ratio may reflect the level of immune function of an individual [18].

Heat shock proteins (HSPs), implicated in a wide range of physiological functions including immunity [19], can and do exit mammalian cells, interact with cells of the immune system, and exert immunoregulatory effects [20]. In addition, it is also well known that HSP expression levels are low under normal physiological conditions, and that they rise under stressful conditions. In early pregnancy, a high risk of adverse pregnancy outcomes is associated with increased HSP70 levels [21]. Whether the expression of placenta HSP70 changes in a low-temperature environment during pregnancy has yet to be shown. We therefore determined to examine placentas from cold-stressed and non-cold-stressed dams. Our findings indicate that prenatal cold stress leads to a significant increase in placental expression of HSP70.

HSP70, an important endogenous ligand, can utilize CD14/TLR2/TLR4 to induce proinflammatory cytokine production via the MyD88/NF-κB signal pathway [20]. The cytokines synthesized as a result, including TNF-α, IL-6, IL-12, and IL-1β, are in turn known to activate NF-κB [22]. Prenatal stress leads to dysbiosis, which is associated with increased IL-1β, both in the placenta and in the brain of female fetuses [23]. In the current study, we found a significant increase in female placental expression of IL-1β and IκBα under conditions of cold stress. NF-κB induced IκBα expression provides a robust and dominant negative feedback loop [24]. Negative feedback control is essential in regulating NF-κB activation, as it leads to transcriptional upregulation of the IκBα and p105 genes, since both of these genes have NF-κB responsive elements in their promoters [25, 26]. This might indicate that the enhanced IκBα expression occurs through a negative feedback mechanism. We hypothesize that the HSP70/TLR4/NF-κB pathways play a key role in prenatal cold stress.

The hypothalamic–pituitary–adrenal (HPA) axis regulates the adrenal synthesis of GCs and their release into the systemic circulation. Under normal conditions, endogenous GC levels in serum display circadian variations, but under a variety of physical and psychological stresses, levels of endogenous GCs are strongly elevated, due to the influence of the hypothalamus [27]. During gestational cold stress, plasma corticosterone levels increased from day 15 of gestation, and remained high until the end of gestation [28]. In the present study, we also found high corticosterone levels during gestational cold stress, and observed an increase in placental GR and MR in response. Although GCs have been reported beneficial in promoting fetal lung maturation, overexposure to GCs may have effects, either directly or indirectly, upon the developing fetus [29, 30]. GCs exert their effects through binding to GR, a ligand-dependent transcription factor [31], in both genomic and non-genomic ways, in almost every tissue in the human body. While most GCs are mediated through binding to the widely expressed GR, GCs can bind with even greater affinity to the MR [32]. In the absence of GCs, GR resides in the cytoplasm, bound to chaperone HSP90 and HSP70 [33]. The presence of a functional GR during gestation is essential for postnatal survival as well as during fetal development [29]. High corticosterone levels may induce increased expression of GR and MR during gestational cold stress. We observed that gestational cold stress induce an increase in IRF3 — a downstream effector of TLR3/4, and an essential activator of several IFN and chemokine genes. GR is able to interact with, and alter the transcriptional activity of IRF3, which binds to DNA-bound regulator-NF-κB, utilizing p160 GRIP1 as a corepressor [34].

The availability of natural glucocorticoids in tissues is regulated by locally expressed 11β-HSD [27]. The effect of glucocorticoids, signaling through MR and GR, is often dampened by the local co-expression of the glucocorticoid inactivating enzyme 11β-HSD2 [32, 35]. 11β-HSD2 is one type of 11β-HSD that abundantly present in the placenta and protects the fetus from excessive maternal glucocorticoids. The biologically active glucocorticoid is unbound cortisol that can be converted to the inactive form, cortisone, by 11β-HSD2 [36]. Under normal conditions, rat placental 11β-HSD2 is produced later in gestation, resulting in less loss of glucocorticoid activity [30], to allow maternal glucocorticoids to stimulate late fetal maturation. The results of this study indicate a high expression of 11β-HSD2 in placental tissue during gestational cold stress. Prenatal cold stress induced high endogenous corticosterone levels, in which case a feedback mechanism attempted to increase 11β-HSD2 expression to protect the body from damage. The effect of GCs on the regulation of placental 11β-HSD2 is still controversial: maternal betamethasone administration dramatically increased 11β-HSD2 mRNA and protein levels in baboons [37], while high cortisol levels decrease 11β-HSD2 enzyme activity in sheep in late pregnancy [38].

Caspase 3 was the main executors of apoptosis, and the activation is a central event in apoptosis, it was a key enzyme in the mitochondria-dependent apoptosis pathway [39]. Bcl-xL (anti-apoptotic proteins) and Bax (pro-apoptotic proteins) belong to the Bcl-2 family. Bcl-xL binds Bax at mitochondria, inhibiting Bax oligomerization, and thus, apoptosis [40]. It has been demonstrated that cold stress causes severe liver damage due to decreased levels of glutathione (GSH) and increased protein carbonylation, lipid oxidation, and abundance of intracellular reactive oxygen species (ROS), leading to oxidative stress-induced apoptosis in the liver. In our previous study, Hsp70 suppressed apoptosis of buffalo rat liver (BRL) cells by regulating the expression of Bcl-2, cytochrome C, and caspase 8/3 [41]. Cold stress may affect placental apoptosis. We examined the expression of the apoptosis-associated Bcl-xL, Bax and Caspase-3 protein in placentas. We observed that gestational cold stress induced high HSP70 levels, and that the ratio of Bcl-xL to Bax expression reached a maximum when cold stress lasted 5 d and 7 d, and a high expression of Caspase-3 after cold stress. The balance between the expression of the antiapoptotic gene Bcl-2 and the proapoptotic gene Bax is considered a good indicator of apoptotic activity [42]. Gestational cold stress induce placental apoptosis; however, over time, negative feedback mechanisms increase the expression of Bcl-xL and decrease that of Bax, inhibiting placental apoptosis.

In addition to the above experiments, neonates’ plasma GH levels were measured at postnatal days 7 and 25. Interestingly, gestational cold stress provoked a decrease in neonates’ plasma GH levels. Abnormalities in the GH-IGF axis are commonly described in growth-retarded fetuses and neonates, as well as in many adult diseases associated with low birth weight [43]. Previous research has suggested that prenatal stress-induced increases in maternal glucocorticoids impairs the development of the adult offspring’s glucocorticoid response [44]. Acute stress inhibits GH secretion in rats [45]. Glucocorticoids can cross the placenta, weaken the HPA axis, which mediates the bodily responses to stress, and reduce pituitary GH secretion, affecting fetal development.

**Figure 7:**
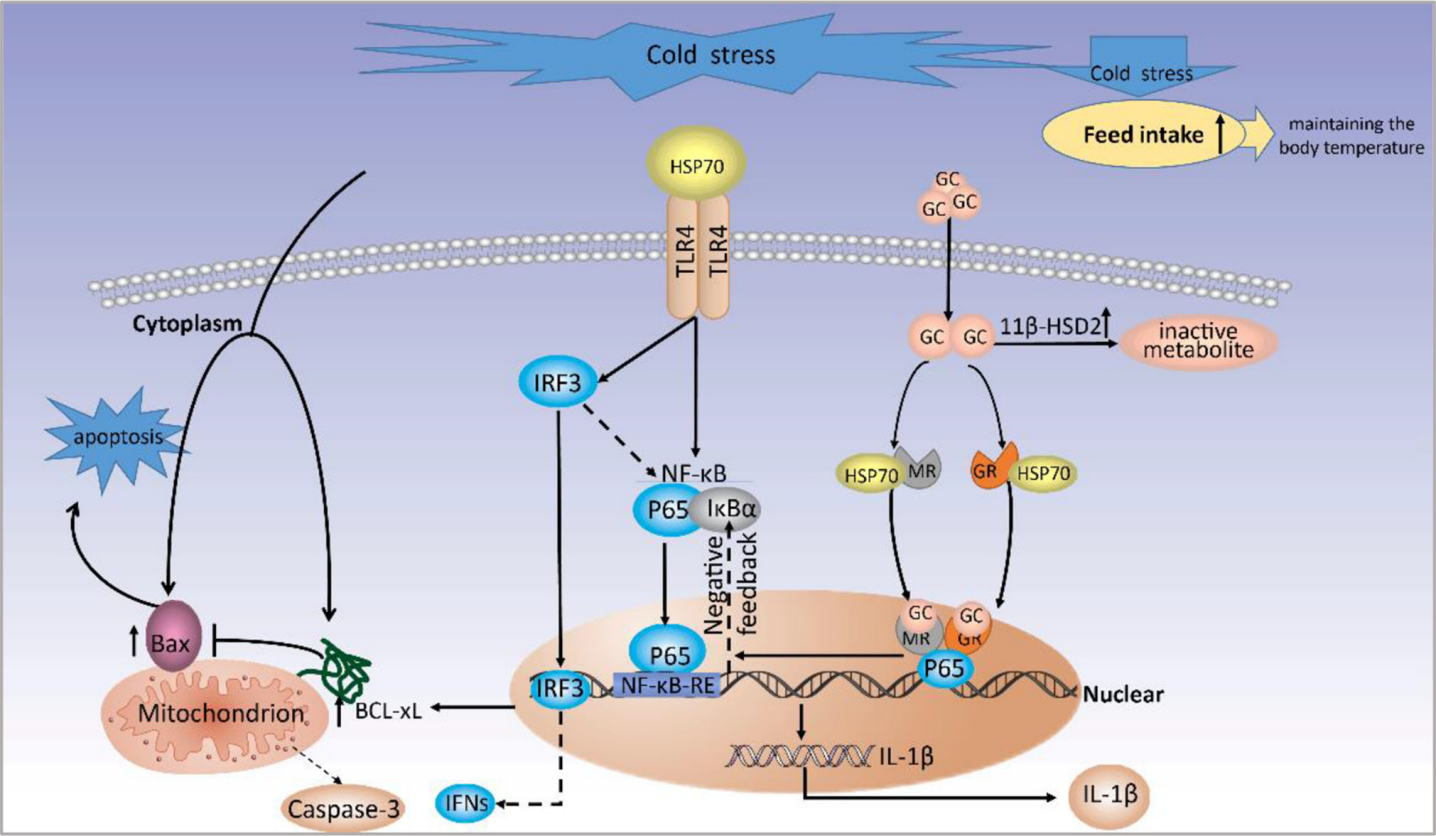
A proposed model for molecular mechanisms of prenatal cold stress on placental

In summary, the present study demonstrated that prenatal cold stress in rats induces inflammation and apoptosis in the placenta, and results in low plasma GH levels, and the role of prenatal cold stress on placental was demonstrated (Figure 7). We will further examine the influence of gestational cold stress on offspring in a future study, providing a better understanding of the role of prenatal cold stress in the development of offspring.

## MATERIALS AND METHODS

### Animals and ethics

30 male (280 ± 20 g) and 60 female (230 ± 20 g) specific pathogen free (SPF) Wistar rats were obtained from the Experimental Animal Center, Qiqihar Medical University, and kept in a climatic chamber at an ambient temperature of 22 ± 2°C and 40 % relative humidity, under a 12/12 h light/dark cycle (light on from 8:00 a.m. to 8:00 p.m.), and with free access to food and water. Procedures involving animals were approved by the Animal Care Committee of the Heilongjiang Bayi Agricultural University (Daqing, China), and all the experiments protocols were performed in accordance with relevant guidelines and regulations of the Heilongjiang Bayi Agricultural University (Daqing, China). Animals were allowed to acclimate for at least one week before being subjected to experimental conditions.

### Prenatal stress and sample collection

Vaginal smears were taken daily from female rats to determine the phase of the estrus cycle prior to mating. On the day of proestrus, two females were placed with one male rat at 8:00 p.m. and vaginal smears were checked for the presence of sperm before 8:00 a.m. the next morning. The day on which a vaginal smear with sperm cells was obtained was designated as gestational day (GD) 0 [46]. Pregnant rats were individually housed in polypropylene cages (37.5×27.3×16.5 cm) at a temperature of 22 ± 2°C for GD0–14. At GD14, they were randomly allocated to either stressed (n = 30) or non-stressed (control, n = 30) treatment conditions. According to the number of stress days, stressed and control groups were further divided into 1d (GD15), 3d (GD17), 5d (GD19), 7d (GD21), and Term Delivery (TD) groups (n = 6 in each), TD groups were for the pups analysis. Non-stressed pregnant rats were performed at 22 ± 2°C. Stressed pregnant rats were exposed to cold stress, performed at 4°C in a climatic chamber. Pregnant rats were anesthetized on GD15, GD17, GD19, and GD21 with pentobarbital sodium and euthanized for sample collection. After the parturition of TD groups, the stressed as same as the non-stressed groups were transferred to a climatic chamber at a temperature of 22 ± 2°C for the pups analysis. Blood samples were collected from the anesthetized pregnant rats on GD15, GD17, GD19, and GD21. Fetus-placentas were obtained from the mid-region of each uterine horn (total of six per mother). Labyrinth placental zones which was the major site of maternal-fetal transfer and undergoes rapid growth over the final week of gestation to support the growing fetus were separated [47], and the samples of per mother were mixed. On postnatal days (PD) 7 and 21, offspring of each group were anesthetized with pentobarbital sodium and euthanized. Blood samples were collected from the offspring. All samples were frozen in liquid nitrogen and stored at −80°C for subsequent examination.

### Body weight, feed intake and rectal temperature

Between GD14 and GD21, maternal body weight, feed intake, and rectal temperature were taken daily. The body weights of offspring were also taken daily from PD1–21. The total weight gain was calculated at the end of the experimental period.

### Maternal plasma CORT and neonatal GH levels

The levels of growth hormone (GH) and corticosterone (CORT) in plasma and amniotic fluids were detected using ELISA kits according to the manufacturer’s protocol (both R&D Systems, USA).

### Flow cytometry analysis of blood CD4, CD8

After stressed 1d, 3d, 5d and 7d, 100 μl samples of anti-coagulated whole blood were collected. The whole blood was then stained with FITC anti-rat CD4 (#201505, Biolegend, USA) and PE anti-rat CD8a (#200608, Biolegend, USA) for 15–20 min, while shielded from light at room temperature of 22 ± 2°C. After incubation, the samples were fixed with 2ml 1X red blood cell (RBC) lysis buffer (#420301, Biolegend, USA) for 10 min, while shielded from light at room temperature, before being centrifuged at 350*g* for 5 min. The resulting supernatant was discarded, and cells were washed and resuspended in at least 2 ml of cell-staining buffer for flow cytometry analysis (Beckman CytoFLEX, USA).

### Protein extraction and western blot

Tissue samples were homogenized in RIPA buffer (#P0013B, Beyotime, China), and their protein concentration was determined using a BCA protein assay (#P0010, Beyotime, China), then separated by SDS-PAGE and transferred onto PVDF membranes (0.22 μm, 0.45 μm, Millipore, Germany). PVDF membranes were blocked in TBST (Tris-buffered with 0.1 % Tween-20) with 5 % skim milk, and the membrane was incubated with primary antibodies at 4°C overnight. Signals were detected using the ECL kit (#P0018, Beyotime, China). Blots were imaged using the ChemiDoc XRS (Bio-Rad) and analysis of western blot images was performed using ImageJ software (http://imagej.nih.gov/ij/). Beta-actin was used as the reference protein.

HSP70 (1:1000, #ab47455) and Bcl-xL (1:10000, #ab178844) antibodies were purchased from Abcam (Amyjet Scientific Inc, UK). Bax (1:3000, #50599-2-lg), β-actin (1:15000, #60008-1-lg), P65 (1:1000, #10745-1-AP), GR (1:2000, #24050-1-AP), IL-1β (1:1000, #16806-1-AP), MR (1:2000, #21854-1-AP), 11β-HSD2 (1:500, #14192-1-AP), TLR4 (1:2000, #19811-1-AP), IRF3(1:2500, #11312-1-AP), IκBα (1:2500, #10268-1-AP), Caspase-3 (1:500, #19677-1-AP) and HSP90 (1:5000, #13171-1-AP) antibodies were purchased from Proteintech (USA). Secondary antibodies were labeled with horseradish peroxidase goat anti-mouse IgG (1:8000, # SA00001-1, Proteintech, USA) and goat anti-rabbit IgG (1:8000, #SA00001-2, Proteintech, USA).

### Statistical analysis

Data were expressed as mean ± standard deviation from the mean (SD). Two-way ANOVA for repeated measures was used to analyze data from maternal body temperature, food intake, body weight gain, plasma CORT levels, pubs’ plasma GH levels and pubs body weight. For placental western blot parameters, comparison between groups was performed using Student’s t-tests. The data were analysed using GraphPad Prism Software (La Jolla, CA, USA). Statistical significance was declared at *P* < 0.05.

## SUPPLEMENTARY MATERIALS FIGURES AND TABLES


