# Supplementary material for: Impact of prenatal cold stress on placental physiology, inflammatory response, and apoptosis in rats
Source: Oncotarget. 2017 Dec 14;8(70):115304–14. doi: 10.18632/oncotarget.23257 (PMC5777773; doi:10.18632/oncotarget.23257)
Supplement: Supplementary file 1 [file oncotarget-08-115304-s001.pdf]

Impact of prenatal cold stress on placental physiology, inflammatory response, and apoptosis in rats

SUPPLEMENTARY MATERIALS

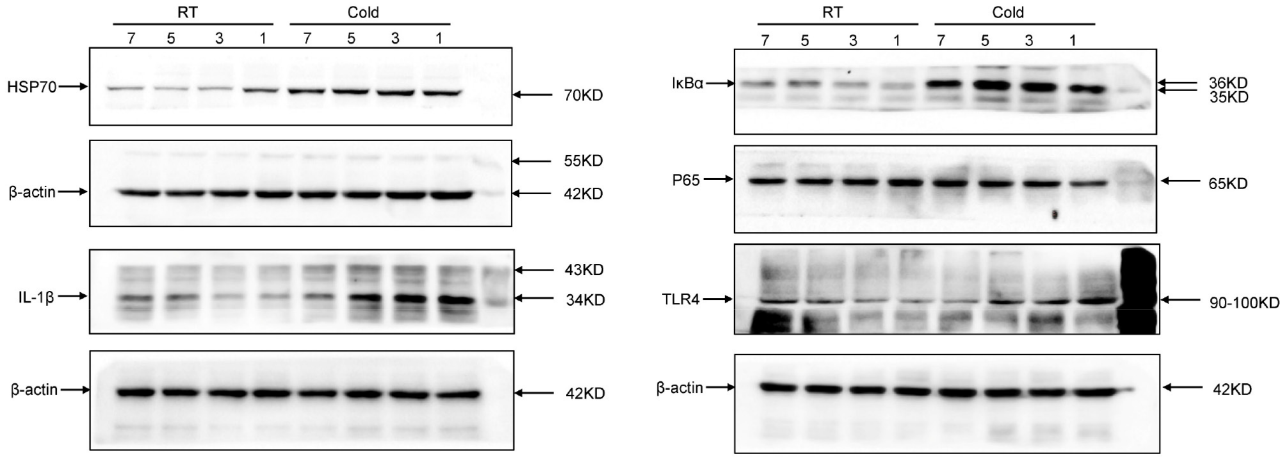

Supplementary Figure 1: Impact of prenatal cold stress on the activation of the HSP70/TLR4/NF-κB signaling pathway in placenta. Full length blots of cropped images shown in Figure 3. Position of bands for the protein molecular weight makers are indicated to the right of the blots.

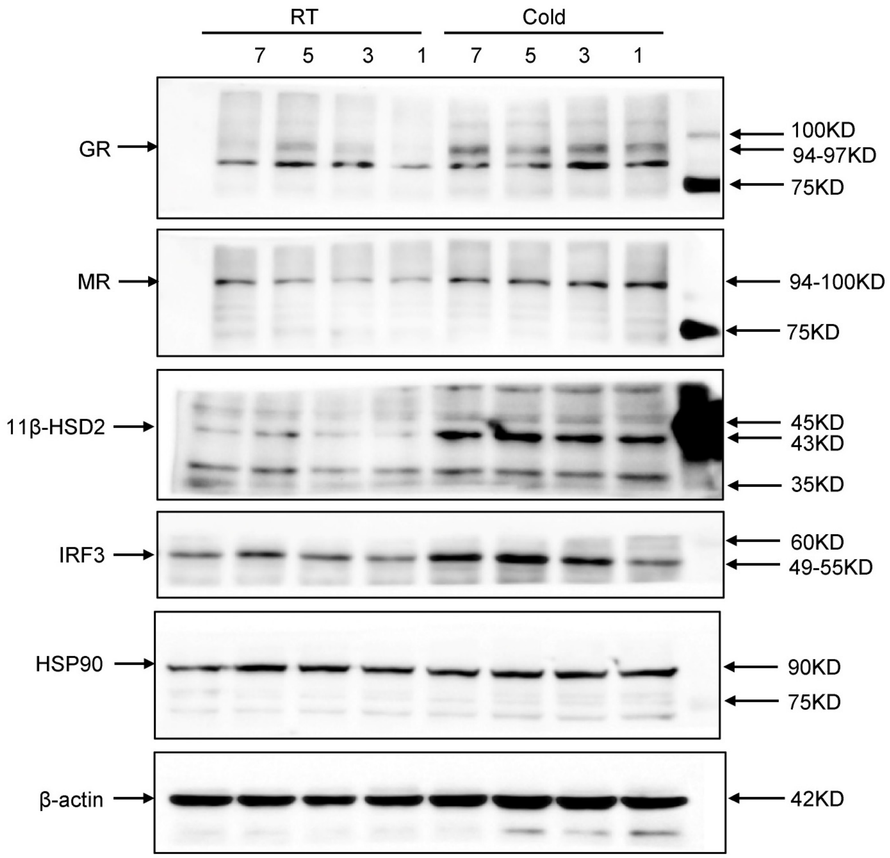

Supplementary Figure 2: Effect of prenatal cold stress on the expression of GR., MR, 11β-HSD2, and IRF3 proteins. Full length blots of cropped images shown in Figure 4. Position of bands for the protein molecular weight makers are indicated to the right of the blots.

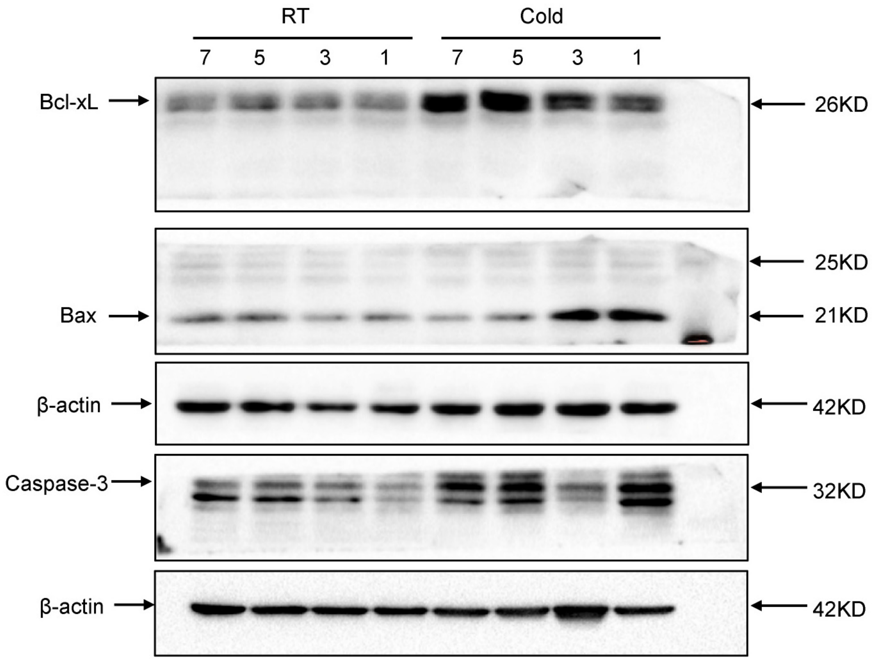

Supplementary Figure 3: Effect of prenatal cold stress on the expression of Bcl-xL, Bax and Caspase-3 proteins. Full length blots of cropped images shown in Figure 5. Position of bands for the protein molecular weight makers are indicated to the right of the blots.
